# Attachment of Respiratory Pathogens and *Candida* to Denture Base Materials—A Pilot Study

**DOI:** 10.3390/jcm12196127

**Published:** 2023-09-22

**Authors:** Anne Schmutzler, Catalina Suzana Stingu, Elena Günther, Reinhold Lang, Florian Fuchs, Andreas Koenig, Angelika Rauch, Sebastian Hahnel

**Affiliations:** 1 Department of Prosthetic Dentistry, Regensburg University Medical Center, 93042 Regensburg, Germany; 2 Institute for Medical Microbiology and Virology, Leipzig University Clinics, 04103 Leipzig, Germany; 3 Department of Prosthetic Dentistry and Dental Materials Science, Leipzig University, 04103 Leipzig, Germany

**Keywords:** biofilm, PMMA, PEEK, *Candida*, denture, prosthesis

## Abstract

Denture prostheses are an ideal and extensive reservoir for microorganisms to attach to their surfaces. The aim of the study was to elucidate interactions between materials for the fabrication of denture bases and the attachment of microorganisms, focusing on respiratory pathogens and *Candida* species. Specimens (6 mm × 1 mm) with a standardized surface roughness (Sa = 0.1 µm) were prepared from heat-pressed polymethyl methacrylate (PMMA), CAD/CAM-processed PMMA, and CAD/CAM-processed polyether ether ketone (PEEK). The specimens were randomly placed in the vestibular areas of complete upper dentures in seven patients and were removed either after 24 h without any oral hygiene measures or after a period of four weeks. The microorganisms adherent to the surface of the specimens were cultivated and subsequently analyzed using mass spectrometry (MALDI-TOF). The means and standard deviations were calculated, and the data were analyzed using a two-way analysis of variance (ANOVA) and Tukey post-hoc test where appropriate (α = 0.05). There was a significant increase (*p* ≤ 0.004) in the total bacterial counts (CFU/mL) between the first (24 h) and the second (four weeks) measurements. Regarding quantitative microbiological analyses, no significant differences between the various materials were identified. Respiratory microorganisms were detected in all samples at both measurement time points, with a large variance between different patients. Only after four weeks, *Candida* species were identified on all materials but not in all participants. *Candida* species and respiratory microorganisms accumulate on various denture base resins. While no significant differences were identified between the materials, there was a tendency towards a more pronounced accumulation of microorganisms on conventionally processed PMMA.

## 1. Introduction

Data gathered from a cross-sectional study in France indicate that almost 14% of the adult population is supplied with removable dental prostheses (RDPs) [1]. Especially in the older age groups, edentulism and complete dentures are still very common [2]. Due to current demographic developments, the number of people over the age of 60 is expected to increase to two billion by 2050 [3]. Thus, despite of the implementation of frequent and effective preventative dental check-ups, dental prophylaxis, and an associated decline in edentulism [4], it can be expected that complete dentures will continue to be a relevant treatment option in prosthetic dentistry.

Complete dentures are in close contact with the oral mucosa and feature an extensive interface. This circumstance makes complete dentures an ideal reservoir for oral biofilms, which have been defined as a “complex, functional community of one or more species of microbes, encased in an extracellular polysaccharide matrix and attached to one another or to a solid surface” [5]. During the formation of biofilms, a salivary pellicle consisting of proteins, lipids, and carbohydrates initially forms on the previously cleaned surface within hours as a result of electrostatic interactions. The components of the pellicle act as receptors for the attachment of microorganisms, which are referred to as initial or pioneer colonizers. These mainly include Gram-positive streptococci (e.g., Streptococcus mitis/oralis/sanguinis) and rods. During the maturation process of the biofilm, further bacteria—so-called late colonizers—attach themselves to the biofilm via co-adhesion. Subsequently, there is a progressive development of the biofilm, which finally consists of facultatively and obligatory anaerobic organisms, Gram-negative cocci and rods, fusobacteria, spirochetes, and actinobacteria [6,7]. So far, about 700 bacterial [8] and up to 100 fungal species [9] have been identified in the oral microbiome. The human body and the microbiome form a symbiotic community called holobiont [10]. Environmental factors influence this dynamic relationship, which can lead to changes in the oral microbiome. Disturbances in the balance between microorganisms and the immune system may affect oral health [10]. The conventional wisdom is that oral biofilms can cause and promote local diseases such as gingivitis, periodontitis, oral candidiasis, dental caries, and endodontic infections, as well as systemic diseases such as aspiration pneumoniae and blood-borne infections.

With regard to these considerations, it has been highlighted that between 11% and 70% of all patients wearing RDPs suffer from denture stomatitis [11]. *Candida albicans* has been identified as the major causative microbial agent in denture stomatitis [12] and can be found in different morphological forms: blastophores, hyphae, and pseudohyphae. The maturation of oral biofilms causes an increased prevalence of the hyphae form, which is associated with increased virulence and pathogenicity [13,14].

In addition to that, it was reported that wearing RDPs is a risk predictor for an increased incidence of pneumonia [15]. It was shown that in older people wearing complete dentures during sleep, the risk for pneumonia increases by a factor of 2.3 [16]. The main reason for this phenomenon is the accumulation of biofilms on the RDP, which may contain respiratory pathogens. While *S. pneumonie* is regarded as the most common causative agent of pneumonia [17], other potential respiratory pathogens such as *S. aureus*, *P. aeruginosa*, *H. influenza serotype B*, *S. pyogenes*, and *M. catarrhalis* have also been identified in healthy denture wearers [18]. The accumulation of biofilm is promoted by inadequate or lacking oral hygiene [19]. Dysphagia and chronic obstructive pulmonary disease may further increase the risk for aspiration pneumonia [20]. 

A simple way to prevent the onset of these diseases is regular and sufficient denture cleaning. A mechanical cleaning of RDPs can be performed with brushes, microwave irradiation, and ultrasonic devices. Chemical cleaning agents include products based on sodium hypochlorite (NaOCl), peroxides, neutral peroxides with enzymes, enzymes, or acids. Combinations of mechanical and chemical protocols can also be employed, and are superior to the application of sole mechanical or chemical protocols [21]. However, tactility and visual ability usually decrease with increasing age. Thus, a sufficient cleaning of RDPs may be difficult for impaired patients and, in some cases, may also not be performed without help. In nursing homes, the staff are often restricted with time, which is why denture hygiene is regularly not adequately performed [22].

For the fabrication of RDPs, several materials are available on the dental market, including polymethyl methacrylate (PMMA) and polyether ether ketone (PEEK). In the dental laboratory, these materials can be processed by heat pressing (PMMA) or with computer-aided design/computer-aided manufacturing (CAD/CAM) techniques by milling from pre-polymerized blanks (both PMMA and PEEK). Pre-polymerization under industrial conditions produces materials with improved stability and mechanical behavior as well as less accumulation of biofilms [23]. With regard to microbial adherence, various surface properties of dental materials may have an impact, including surface roughness, surface free energy, and—to a minor extent—surface charge. With regard to surface roughness, the conventional wisdom is that rough surfaces favor the accumulation of microorganisms as they provide larger surface areas that are available for microbial attachment than smooth surfaces [24]. Moreover, rough surfaces protect microorganisms from oral shear forces during their initial attachment and may also impair the removal of organized microbial biofilms [25]. The interactions between surface free energy and the attachment of microorganisms are complex, and simple correlations have not yet been established [25]. However, it was summarized from the existing evidence that bacteria preferentially attach to surfaces with a high surface free energy [26,27]. Schmalz and Cieplik [28] and Teughels et al. [29] have provided comprehensive overviews dealing about the explanatory models. 

Since the biofilms on RDPs may affect systemic health, it is worth developing materials that prevent the attachment of microorganisms such as respiratory pathogens and *Candida albicans* [30]. As no clinical data are currently available regarding the impact of the denture base material on the attachment and proliferation of respiratory microorganisms, the aim of the current study was to elucidate potential interactions between various denture base materials and the attachment of several microorganisms associated with respiratory diseases as well *Candida albicans*. It was hypothesized that resin-based materials polymerized under industrial conditions show less accumulation of these particular microorganisms and *Candida* species than conventional resin-based materials for the fabrication of RDPs. 

## 2. Materials and Methods

**Figure 1:**
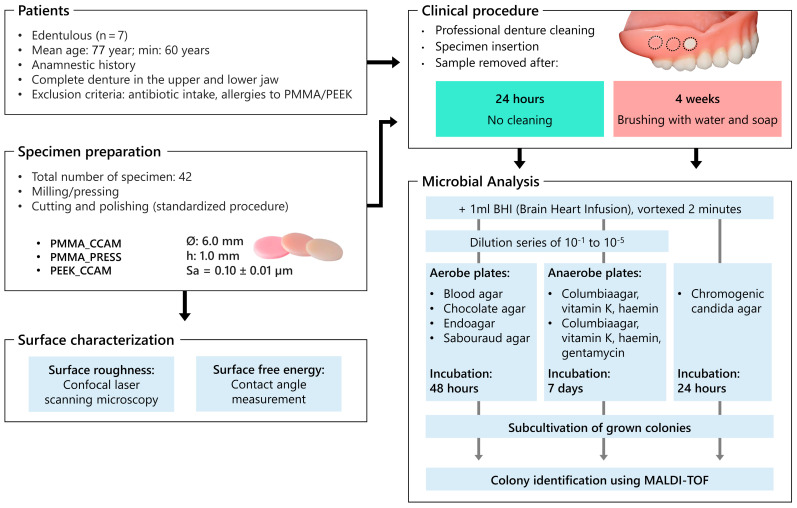
Study design; CAD/CAM-processed polymethyl methacrylate (PMMA_CCAM); heat-pressed polymethyl methacrylate (PMMA_PRESS); CAD/CAM-processed polyether ether ketone (PEEK_CCAM); number (n); diameter (ø); height (h); arithmetical mean height of surface roughness (Sa); matrix-assisted laser desorption ionization time-of-flight mass spectrometry (MALDI-TOF).

The design of the study is displayed in Figure 1.

### 2.1. Study Population

Seven edentulous patients (57.1% female) with a minimum age of 60 years (mean age 77.3 years ± 7.1 years; 72–92 years) supplied with complete dentures fabricated from polymethyl methacrylate in the upper and lower jaw were included in this observational study, which was performed between March 2020 and March 2022 at the Department of Prosthetic Dentistry and Dental Materials Science of the Leipzig University. All patients were able to carry out oral hygiene measures on their own accord; only patients with adequate oral hygiene were included. The grading of prosthetic hygiene took place by visual assessment. Care was taken to ensure that no plaque was visible on the dentures; only little amounts of stain were tolerated. The exclusion criteria included the intake of antibiotics in the last six months as well as allergies to PMMA or PEEK. Information on diseases such as bronchitis, pneumonia, and nasogastric/tracheal intubation within the last six months and pulmonary diseases such as chronic obstructive pulmonary diseases as well as an anamnestic history of tuberculosis (ever) were recorded. The patients were informed in advance about the investigation and consented in writing to participate in the study that had been approved by the local Ethical Committee (071/19-ek). 

### 2.2. Specimen Preparation 

Forty-two cylindrical specimens (Ø: 6 mm, h: 1 mm) were prepared from polymethyl methacrylate either through heat pressing (Ivobase Hybrid, Ivoclar Vivadent, Schaan, Liechtenstein) (PMMA_press) or CAD/CAM (Vita Vionic Base, Vita, Bad Säckingen, Germany) (PMMA_CCAM), and polyether ether ketone was prepared through CAD/CAM (Juvora, Invibio Ltd., Thornton-Cleveleys, UK) (PEEK_CCAM). The surfaces of the specimens were cut (IsoMet 4000, Buehler, Lake Bluff, IL, USA) and ground/polished using a semi-automatic grinding machine (Pedemin-2/DAP-V, Struers, Willich, Germany) with silicon carbide paper (up to P2000) and a composite disc (MD-Largo, Struers, Willich, Germany) with diamond polishing suspension (grain size: 15 μm). Consistent surface roughness was verified through confocal laser scanning microscopy. Subsequently, all specimens were stored under light-proof conditions in distilled water for six days at 37 ± 1 °C to minimize the impact of residual monomer leakage on cell viability. The specimens were cleaned with ethanol (70%) and applicator brush tips prior to being mounted onto the dentures. 

### 2.3. Surface Characterization 

Sample surfaces were validated with confocal laser scanning microscopy (Keyence VK-X1000/1050, Keyence, Osaka, Japan) with a 50× objective (Nikon CF IC EPI Plan 50×; NA: 0.5; NIKON, Osaka, Japan) and a red laser (λ  =  661 nm). Five areas per sample were analyzed using the software “MultiFileAnalyzer” 2.1.3.89 (Keyence, Osaka, Japan), according to ISO 25178-2:2012 and appropriate filtering (S-Filter: 0.5 µm; F-Filter: 0.1 mm; Filter type: spline; end effect correction). For an analysis of the surface texture, the parameters of the arithmetical mean height (Sa), developed interfacial area ratio (Sdr), the auto-correlation length (Sal), and the skewness (Ssk) and the kurtosis (Sku) of the surface distribution were further surveyed.

The analysis of the surface free energy was performed on 15 samples via contact angle measurements. For this purpose, 0.5 µL each of purified water and diiodomethane was applied to the surface using a “DSA25S” with “Liquid Needle DO3252” and measured using “ADVANCE 1.11” (all KRÜSS, Hamburg, Germany) after a delay of 30 s (fitting method: ellipse). Following the advanced contact angle measurements, another 0.5 µL was added to the drops and measured again until a total volume of 2 µL was reached [31]. The total surface free energy as well as the polar and dispersive parts were calculated according to Owens and Wendt [32]. 

### 2.4. Specimen Insertion 

One specimen of each material was randomly placed in the vestibular area of the upper denture molars according to a previously established randomization list to counteract known and unknown confounding variables. The specimens were fixed with a soft denture liner (Mucopren soft, Kettenbach, Eschenburg, Germany) in a small cavity (Ø: 6.5 mm, h: 1 mm) that had previously been inserted using a trepan drill. Prior to mounting the specimens, the upper and lower RDPs were forwarded for professional cleaning and polishing to remove adherent biofilms. 

### 2.5. Study Protocol

First study part: The patients wore the RDPs equipped with the various specimens permanently for 24 h without any hygiene interventions. The specimens were then carefully removed, placed in an Eppendorf tube with 1 mL of brain heart infusion (BHI) bouillon, and immediately forwarded for microbiological analysis. The RDPs were professionally cleaned and polished. 

Second study part: The RDPs were equipped with a new set of specimens. The patients wore the RDPs for 24 h/day and were instructed to clean the dentures daily by brushing with water and neutral soap as demonstrated. For this purpose, a denture brush was moistened with neutral soap, and the denture was then cleaned schematically from the denture base to the occlusal surface. The patients were instructed to refrain from the application of chemical detergents as well as antiseptic agents. After a period of four weeks, the specimens were carefully removed and forwarded for microbial analysis accordingly. 

Group one (24 h—no cleaning) was introduced to produce biofilms on the specimens undisturbed from hygiene measure. The period of 24 h may represent a reasonable point between the initial adhesion and the maturation of biofilm and was chosen according to previous studies [33,34,35]. A period of four weeks (Group two) was introduced to produce biofilms on the surface of the specimens that occur during continuous and regular hygiene regimens.

### 2.6. Microbial Analysis 

Specimens immersed in BHI (brain heart infusion) broth were vortexed for 2 min (IKA VF2 Vortex Mixer, IKA^®^-Werke, Staufen, Germany); subsequently, a dilution series of 10^−1^ to 10^−5^ was prepared. A volume of 0.1 mL of each dilution was inoculated onto seven different culture media (Figure 1) using a pipette (Multipette^®^ (4780); Eppendorf Combitips advanced^®^, Eppendorf, Hamburg, Germany) and sterile disposable spatulas in a microbiological safety workbench (HeraSafe KS Class II Workbench, Thermo Fisher Scientific, Dreieich, Germany). The media blood agar, chocolate agar, Endoagar, and Sabouraud agar were aerobically incubated for 48 h at 37 °C with approximately 5% CO_2_ in a CO_2_-incubator (Heracell 150i CO_2_-Incubator, Thermo Fisher Scientific, Dreieich, Germany). The chromogenic *Candida* agar plates were incubated aerobically at 36 °C for 20 h. The Columbia blood agar plates were cultivated for seven days in an anaerobic workstation (Whitley MG 1000, anaerobic workstation, Meintrup Laborgeräte, Lähden, Germany) at 37 °C. All different colonies were visually described, counted, and subcultivated. After re-incubation (aerobe: 24 h; anaerobe: 4–7 d), colonies were identified using matrix-assisted laser desorption ionization time-of-flight mass spectrometry (MALDI-TOF; VITEK^®^ MS, bioMérieux, Lyon, France), employing the commercially available V2.0 knowledge database for clinical use. Chromogenic *Candida* agar plates were used in addition to the Sabouraud agar plates for the easier identification of fungal species. All microbiological analyses were performed by the same examiner. 

### 2.7. Statistical Analysis

The means and standard deviations for colony forming units per mL (CFU/mL) were calculated. The normal distribution of the data was assessed using the Shapiro–Wilk test and the equality of variance was evaluated using Levene’s tests. A two-way analysis of variance (ANOVA) and the Tukey post-hoc test were applied where appropriate. The level of significance (α) was set to 0.05. All analyses were performed with IBM SPSS Statistics (Version 28.0). 

## 3. Results

**Figure 2:**
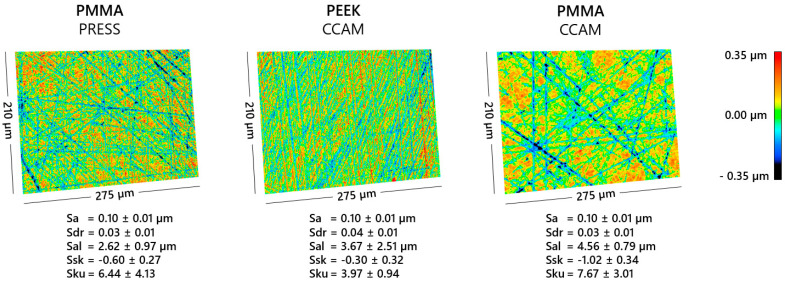
Exemplary three-dimensional surface renderings of the used materials with the corresponding surface texture parameters of the respective sample group; CAD/CAM-processed polymethyl methacrylate (PMMA_CCAM); heat-pressed polymethyl methacrylate (PMMA_PRESS); CAD/CAM-processed polyether ether ketone (PEEK_CCAM); arithmetical mean height (Sa); developed interfacial area ratio (Sdr); auto-correlation length (Sal); skewness (Ssk); kurtosis (Sku).

After the preparation of the specimens, all the groups resulted in an Sa of 0.10 ± 0.01 µm and an Sdr of 0.03 ± 0.01 to 0.04 ± 0.01 (Figure 2). Based on the three-dimensional surface renderings, the specimens of the PMMA_CCAM group showed a coarser ground pattern with fewer and broader as well as deeper valleys (Figure 2). Correspondingly, a higher Sal with 4.56 ± 0.79 µm and a reduced Ssk with −1.02 ± 0.34 of the surface distribution can be observed for the PMMA_CCAM samples. The Sku is greater than three for all sample groups (3.97 ± 0.94 to 7.67 ± 3.01) but varies in both mean and standard deviation.

Considering the standard deviation, the measurement of the contact angles resulted in almost identical values for the total surface free energy for all groups of specimens with 46.45 ± 2.20 mN/m to 48.17 ± 3.65 mN/m (Table 1). However, the distribution of the polar and dispersive fractions was different, with the former increasing from PEEK_CCAM (11.39 ± 1.92 mN/m) and PMMA_PRESS (8.09 ± 0.94 mN/m) to PMMA_CCAM (13.42 ± 1.57 mN/m) and the latter decreasing from PEEK_CCAM (38.36 ± 1.26 mN/m) and PMMA_PRESS (36.78 ± 1.73 mN/m) to PMMA_CCAM (33.25 ± 1.36 mN/m).

**Figure 3:**
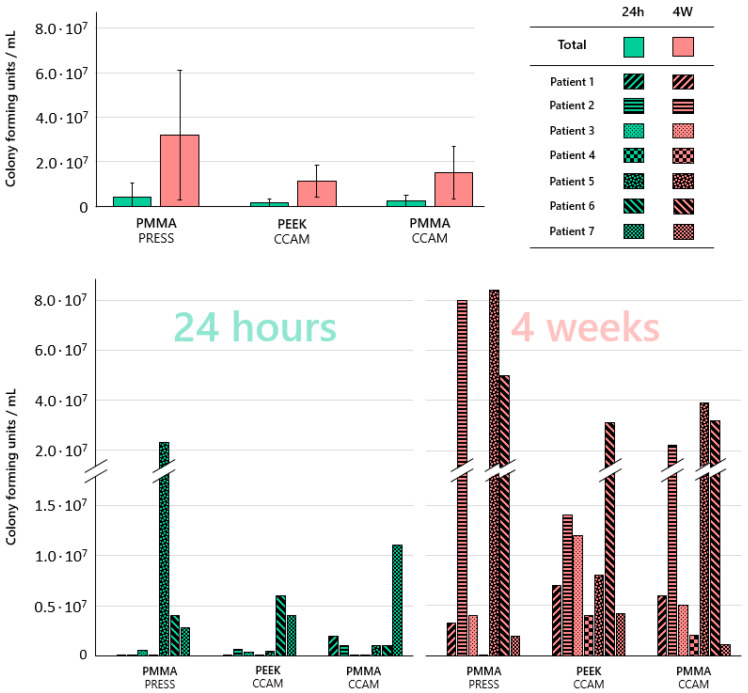
Colony forming units identified on the different materials/in the different participants; CAD/CAM-processed polymethyl methacrylate (PMMA_CCAM); heat-pressed polymethyl methacrylate (PMMA_PRESS); CAD/CAM-processed polyether ether ketone (PEEK_CCAM).

There was significantly more microbial growth on all specimens after four weeks than after 24 h (*p* ≤ 0.004). Higher total bacterial counts as well as larger standard deviations were identified on the specimens fabricated from conventionally processed PMMA than on the other two materials, but no statistically significant differences (*p* ≥ 0.211) between the three materials were determined (Figure 3).

The microbial growth on the different materials in the various patients showed a high variability. Bacteria associated with aspiration pneumonia were identified on the various denture base materials, yet large differences were observed (Table 2 and Table 3). *Candida* species were identified on all materials after four weeks but not after 24 h and not in all participants. 

## 4. Discussion

The results of the current study suggest a rejection of the research hypothesis, as no significant differences in microbial counts were identified in the biofilms grown on the three different resin-based materials for the fabrication of RDPs. Nevertheless, less accumulation of microorganisms on the materials polymerized under industrial conditions than on conventionally processed PMMA was observed. 

The data have to be interpreted within the limitations of the study. These include the design as a pilot study with a limited number of participants, which was due to the corona pandemic that made it particularly difficult to acquire voluntary participants and also to conduct the study in a university hospital setting. Despite the strict inclusion and exclusion criteria, there are still differences between the study participants that can hardly be controlled or standardized. Each patient features a very individual oral microbiome with differences in microbial species as well as microbial load, which might serve as an explanation for the high interindividual variability observed in the current study. The specimens were mounted onto the vestibular areas of the upper denture molars, yet, for higher clinical relevance, it might have been useful to insert the specimens into the basal areas of complete dentures as these are in contact with the oral mucosa. These areas are frequently not polished during the fabrication of the denture and, later, not regularly adequately cleaned. However, the shape of the specimens (flat discs) required an area without pronounced curvatures, and the patients were instructed to use the RDPs as in their common routine, which is why the authors tried to minimize the discomfort associated with the study. Since the fixed specimens were visible to the patients, they were aware of their location. Therefore, an improvement in the thoroughness of the oral hygiene measures during the study cannot be excluded. However, as the improvement in oral hygiene affected all sample bodies, this does not limit the comparability of the results within the study.

Although there were no statistically significant differences between the three materials regarding the total number of microorganisms identified by colonization, the absolute values as well as the standard deviations were higher for conventionally processed PMMA. This observation suggests that the manufacturing method employed for processing the denture base resin may in fact have an effect on biofilm formation. One reason for this phenomenon might be that the industrially standardized production of the materials to be used in a CAD/CAM process generates more homogeneous materials with less porosity and residual monomers in comparison to the conventionally processed denture base resins [36]. With regard to this aspect, it was highlighted that porosities can promote microbial adhesion [37]. While in the current study all specimens were subjected to a standardized polishing protocol prior to being mounted on the RDPs, surface porosities were not investigated, and it might be useful to elucidate a potential correlation between surface porosity and microbial adhesion in further studies. The conventional wisdom is that surface roughness is the most important material-associated predictor for microbial adherence. In order to eliminate the influence of surface roughness on the adherence of microorganisms to the various materials investigated in the current study, all specimens were polished to a Sa value of 0.1 μm, which is far below the commonly accepted threshold of 0.2 μm relevant for microbial adhesion [38]. 

Regarding the amount and effect of the residual monomers eluted from CAD/CAM dental polymers and conventional polymers, conflicting results have been reported. Wei et al. (2022) [39] reported that CAD/CAM dental polymers feature less residual monomer elution and, as a result, higher cell proliferation in cytotoxicity assays than conventional polymers. These results suggest that microbial adhesion to these materials might be enhanced, too [40]. In the current study, all specimens were stored in distilled water prior to the experiments in order to minimize the potential effects of the residual monomer elution. Moreover, it has also been reported that the elution of a residual monomer is more related to the material itself rather than the mode of fabrication [41]. 

The surface texture of the specimens had the same arithmetical mean height (Sa) due to the targeted polishing in order to minimize deviations and their effect on microbial adhesion. Even though primarily the arithmetical mean height was recommended in previous studies for the characterization of biomaterial surfaces [38,42,43], a visible difference in texture could already be observed based on the 3D surface renderings of this study, according to which CAD/CAM-processed PMMA exhibited a more coarse grinding pattern. Specifically, a lower number as well as a greater depth and width of the valleys created by the grinding/polishing process was observed for CAD/CAM-processed PMMA. These features result in a greater auto-correlation length (Sal) as well as in a lower skewness (Ssk) of the surface roughness. The specific surface area of the samples was similar for all samples. The same microbial adhesion on the CAD/CAM-processed PMMA and CAD/CAM-processed PEEK specimens despite the mentioned differences in the surface texture parameters Ssk and Sku in this pilot study confirms the findings of Schubert et al. [43] and Etxeberria et al. [42] that Sa is suitable as a surface characterization of dental biomaterials. Nevertheless, in subsequent studies, a differently pronounced surface texture with the same Sa should be assumed in order to take this into account with respect to any differences that may occur with respect to microbial adhesion. With regard to surface free energy, the results of the current study do not suggest a simple correlation with biofilm formation, which corroborates previously published data from the literature [25]. However, it has to be taken into consideration that the surface free energies between the materials investigated in the current study were rather similar and their relations to biofilm formation might be more pronounced between materials with more distinct surface free energy. 

In the current study, *Candida* species were identified in the biofilms after four weeks but not after 24 h. This result is not surprising as *Candida* species do not belong to the early-colonizing microorganisms in oral biofilms [7]. In addition to these results, the data of the current study emphasize the relevance of RDPs as a potential reservoir of pneumonia-associated microorganisms, as reported by O’Donnell et al. (2016) [18]. Despite the pronounced interindividual variability and strict hygiene interventions during the study, several microorganisms associated with pneumonia were identified in the biofilm on the various materials. The presence of these potential pathogens appears to be independent of the denture base resin. Nevertheless, further studies focusing on potentially pathogenic microorganisms and their presence in biofilms on RDPs are necessary to corroborate the results of this pilot study. 

As a result, the outcome of this study emphasizes that sufficient daily cleaning of RDPs is necessary to avoid microbial colonization. A recently published systematic review from our research group indicates that the combined use of chemical and mechanical denture hygiene interventions is more effective than single cleaning approaches [21]. However, it should be noted that cleaning procedures may cause damage to the denture surface, which may increase surface roughness and affect microbial adhesion. This phenomenon might particularly occur in materials with low surface hardness [44]. Furthermore, the enzymes and acid secreted by microorganisms can also lead to a roughening of the material surfaces, which in turn promotes microbial attachment [45,46]. However, the influence of these aging factors can be considered minor or negligible in the present investigation, as the study period was limited to a period of four weeks.

## 5. Conclusions

The surfaces of RDPs are an ideal reservoir for the accumulation of biofilms. The present study did not find any significant differences in the total bacterial counts on different polymer materials that can be used for the fabrication of RDPs. While no significant differences were identified between the materials, there was a tendency towards a more pronounced accumulation of microorganisms on conventionally processed PMMA. It was shown that the total bacterial counts increase with time. Potential respiratory pathogens as well as *Candida* species were identified on all three examined materials, although there was a large interindividual variability. 

## Figures and Tables

**Table 1:** Measured contact angles and calculated surface free energy of the materials used in the study; CAD/CAM-processed polymethyl methacrylate (PMMA_CCAM); heat-pressed polymethyl methacrylate (PMMA_PRESS); CAD/CAM-processed polyether ether ketone (PEEK_CCAM).

	PMMA_PRESS	PEEK_CCAM	PMMA_CCAM
**Contact angle in °**			
Water	63.89 ± 3.37	69.36 ± 1.81	62.71 ± 2.54
Diiodo methane	45.42 ± 3.22	42.44 ± 2.43	51.83 ± 2.40
**Surface free energy in mN/m**			
Total	48.17 ± 3.65	46.45 ± 2.20	46.67 ± 2.93
Polar part	11.39 ± 1.92	8.09 ± 0.94	13.42 ± 1.57
Dispersive part	36.78 ± 1.73	38.36 ± 1.26	33.25 ± 1.36

**Table 2:** Isolated microorganisms after 24 h; female (f); male (m); CAD/CAM-processed polymethyl methacrylate (PMMA_CCAM); heat-pressed polymethyl methacrylate (PMMA_PRESS); CAD/CAM-processed polyether ether ketone (PEEK_CCAM); colony forming units (CFU).

After 24 h	Patient						
	1 (f)	2 (f)	3 (m)	4 (f)	5 (m)	6 (m)	7 (f)
**Number of different isolated microorganisms per sample**							
PMMA_press	16	6	12	12	13	6	12
PEEK_CCAM	14	7	13	11	14	9	13
PMMA_CCAM	11	5	11	11	13	9	15
**Isolated respiratory bacteria (CFU/mL)**							
*Escherichia coli*							
PMMA_press	3000	0	0	0	0	0	0
PEEK_CCAM	0	0	0	0	0	0	30,000
PMMA_CCAM	0	0	0	0	0	0	0
Enterobacter species							
PMMA_press	0	0	0	0	0	0	0
PEEK_CCAM	1000	0	0	0	0	0	0
PMMA_CCAM	0	0	0	0	0	0	0
Haemophilus influenzae							
PMMA_press	0	0	0	0	1,200,000	0	0
PEEK_CCAM	0	0	0	500	40,000	0	0
PMMA_CCAM	0	0	0	0	60,000	0	0
Klebsiella species							
PMMA_press	0	0	0	0	1,400,000	100,000	0
PEEK_CCAM	0	0	0	0	0	400,000	0
PMMA_CCAM	0	0	0	0	0	300,000	0
Staphylococcus aureus							
PMMA_press	0	0	0	0	7000	0	0
PEEK_CCAM	0	0	0	0	0	0	0
PMMA_CCAM	0	0	0	0	0	0	0
Streptococcus pneumoniae							
PMMA_press	4000	0	0	0	0	0	100,000
PEEK_CCAM	50,000	0	0	1000	50,000	0	0
PMMA_CCAM	120,000	0	0	0	0	0	0
***Candida* species (CFU/mL)**							
PMMA_press	0	0	0	0	0	0	0
PEEK_CCAM	0	0	0	0	0	0	0
PMMA_CCAM	0	0	0	0	0	0	0

**Table 3:** Isolated microorganisms after four weeks; female (f); male (m); CAD/CAM-processed polymethyl methacrylate (PMMA_CCAM); heat-pressed polymethyl methacrylate (PMMA_PRESS); CAD/CAM-processed polyether ether ketone (PEEK_CCAM); colony forming units (CFU).

After Four Weeks	Patient						
	1 (f)	2 (f)	3 (m)	4 (f)	5 (m)	6 (m)	7 (f)
**Number of different isolated microorganisms per sample**							
PMMA_press	15	12	17	4	17	16	16
PEEK_CCAM	15	13	12	16	13	12	13
PMMA_CCAM	14	19	11	16	17	12	15
**Isolated respiratory bacteria (CFU/mL)**							
*Escherichia coli*							
PMMA_press	0	0	0	0	1,000,000	0	2000
PEEK_CCAM	0	0	0	2000	0	0	0
PMMA_CCAM	0	0	0	10,000	110,000	0	0
Enterobacter species							
PMMA_press	0	0	0	0	0	0	20,000
PEEK_CCAM	0	100,000	0	10,000	0	0	0
PMMA_CCAM	0	0	0	30,000	1,100,000	0	0
Haemophilus influenzae							
PMMA_press	0	10,000	0	0	0	0	0
PEEK_CCAM	0	0	0	0	0	0	0
PMMA_CCAM	0	0	0	0	150,000	0	0
Klebsiella species							
PMMA_press	0	0	0	0	0	0	0
PEEK_CCAM	0	0	0	0	0	0	0
PMMA_CCAM	0	0	0	600,000	0	0	0
Staphylococcus aureus							
PMMA_press	0	0	0	0	0	0	0
PEEK_CCAM	0	0	0	0	0	0	0
PMMA_CCAM	0	0	0	0	0	0	0
Streptococcus pneumoniae							
PMMA_press	100,000	0	0	0	0	0	0
PEEK_CCAM	0	0	0	0	0	0	0
PMMA_CCAM	0	0	0	0	0	0	0
***Candida* species (CFU/mL)**							
PMMA_press	0	2,006,000	31,000	0	500,000	3,509,000	0
PEEK_CCAM	0	2,020,000	0	0	0	10,100,000	0
PMMA_CCAM	100	3,140,000	3000	0	0	4,700,000	0

## Data Availability

The datasets generated and/or analyzed during the current study are available from the corresponding author upon reasonable request.
